# Soft tissue constraints on joint mobility in the avian shoulder

**DOI:** 10.1242/jeb.250952

**Published:** 2025-11-10

**Authors:** Oliver E. Demuth, John R. Hutchinson, Vittorio La Barbera, Sharon E. Warner, Daniel J. Field

**Affiliations:** ^1^ Clare College, University of Cambridge, Cambridge CB2 1TL, UK; ^2^ Department of Earth Sciences, University of Cambridge, Cambridge CB2 3EQ, UK; ^3^ Structure and Motion Laboratory, Royal Veterinary College, Hatfield AL9 7TA, UK; ^4^ Museum of Zoology, University of Cambridge, Cambridge CB2 3EJ, UK

**Keywords:** Range of motion, Bird, Ligament, Flight, XROMM, Wing

## Abstract

Joints and their surrounding soft tissues facilitate and restrict vertebrate skeletal motion. Measures of maximal joint mobility provide insight into articular function and its limits on potential joint motion and thereby behaviour. In extinct vertebrates the reconstruction of joint mobility permits us to decipher shifts in locomotor evolution. Such measurements are generally limited to studies of osteological joint mobility. However, only a subset of osteologically feasible poses are biologically feasible because true joint mobility is limited by soft tissues, such as ligaments, that are seldom preserved in the fossil record. To address this issue, we implemented an *in silico* model to simulate avian glenohumeral (shoulder) movement and the constraints imposed by six ligaments on its joint mobility. We evaluated our *in silico* model of the partridge shoulder joint with measured *ex vivo* shoulder mobility using X-ray reconstruction of moving morphology (XROMM). Our results indicate that modelling ligamentous constraints is integral to accurately quantifying shoulder function owing to the role of ligaments in maintaining articular contact during complex glenohumeral motion. Our approach enables more confident estimates of functional joint mobility in both extant and extinct vertebrates and thereby stands to improve inferences of behaviour and musculoskeletal function in the vertebrate fossil record.

## Teaser

**Summary:** Experimental *ex vivo* data and *in silico* simulations shed light on the function of ligaments in the bird (partridge) shoulder joint in maintaining shoulder articulation and constraining joint mobility.

## INTRODUCTION

Studying the functional and articular morphology of joints is crucial for understanding the function of vertebrate locomotor systems. Attempts to reconstruct major evolutionary transformations in locomotor evolution, such as the water-to-land transition among stem tetrapods (Dickson et al., 2021; Pierce et al., 2012) or the origin of avian flight (Heers and Dial, 2012; Pittman et al., 2020) rely on accurate inferences of how extinct vertebrates moved. The articular morphologies of bones may hint at the potential mobility of a joint and how bones move relative to each other in living vertebrates (Manafzadeh, 2023); however, inferences relying solely on these articular morphologies are limited if they ignore the important influence of soft tissues on joint mobility (Holliday et al., 2010; Hutson and Hutson, 2012; Jones et al., 2021; Tsai et al., 2018, 2019).

The mobility of a joint, that is, its range of motion (ROM) and its three-dimensional (3D) envelope, can be estimated by evaluating which joint poses are possible and which are prevented either by bony stops or by joint disarticulation. Joint mobility has often been considered an important foundation for reconstructing locomotor capabilities in extinct tetrapods (Bishop et al., 2023; Demuth et al., 2020; Herbst et al., 2022b; Lee et al., 2023; Nyakatura et al., 2019; Pierce et al., 2012; Richards et al., 2021), with mobility estimates traditionally relying on physical or digital manipulation of individual bones (Senter, 2006; Senter and Robins, 2005). However, the last decade has seen substantial advances in comparative locomotor biomechanics with the introduction of new digital methods (Demuth et al., 2023). These digital toolsets have been adapted from the animation and 3D industry and include novel modelling approaches for musculature (Demuth et al., 2022b; Herbst et al., 2022a), reconstruction of deformed specimens and removal of taphonomic artefacts (Clark et al., 2023; Demuth et al., 2022a; Herbst et al., 2022a; Lautenschlager, 2016), animation and tracking of motion data (Brainerd et al., 2010; Gatesy et al., 2010; Knörlein et al., 2016; Nyakatura and Demuth, 2019; Nyakatura et al., 2019; Wiseman et al., 2022a; Zwafing et al., 2021), and estimation and systematic quantification of joint mobility (Brocklehurst et al., 2025; Griffin et al., 2022; Jones et al., 2021; Manafzadeh and Gatesy, 2020, 2021, 2022; Manafzadeh and Padian, 2018; Regnault et al., 2021; Richards et al., 2021). Such standardised approaches are widely used to systematically investigate joint mobility across all three rotational degrees of freedom (DOF; i.e. rotation around the flexion/extension, abduction/adduction and long axes) and their complex interplay (Brocklehurst et al., 2025; Haering et al., 2014; Kambic et al., 2017). These approaches are therefore an important tool to infer joint function and exclude potential behaviours that would require impossible joint poses (Bishop et al., 2023; Demuth et al., 2020; Gatesy et al., 2009; Griffin et al., 2022; Herbst et al., 2022b,c; Mallison, 2010a,b; Manafzadeh, 2023; Manafzadeh and Padian, 2018; Nyakatura et al., 2015, 2019; Pierce et al., 2012; Richards et al., 2021) under the assumption that these mobility estimates are generally reliable (but see Manafzadeh and Gatesy, 2021) and that only a subset of these poses are/were used in life (e.g. Arnold et al., 2014; Herbst et al., 2022b; Hutson and Hutson, 2012; Kambic et al., 2017; Manafzadeh and Padian, 2018; Manafzadeh et al., 2021). Resultant joint mobility estimates usually form the foundation upon which other parameters, such as behaviour, muscle moment arms or energy expenditure (Baliga et al., 2019; Bishop et al., 2021; Brocklehurst et al., 2022; Cuff et al., 2022; Molnar et al., 2021; Nyakatura et al., 2019; Regnault and Pierce, 2018; Regnault et al., 2021) are investigated. Erroneous exclusions of viable poses or the inadvertent inclusion of physiological inviable poses may, therefore, have repercussions for any downstream analyses, such as the estimation of musculotendon parameters in biomechanical models based upon joint excursion (Bishop et al., 2021; Manal and Buchanan, 2004; Modenese et al., 2016) and/or can lead to erroneous conclusions (Manafzadeh, 2023; Manafzadeh and Gatesy, 2021; Wiseman et al., 2022b).

Recent studies have suggested that simulations that only incorporate three rotational DOFs may not sufficiently capture the true *ex vivo* mobility of a given joint and additional translational DOFs may, therefore, be necessary to incorporate into simulations of joint mobility (Griffin et al., 2022; Lee et al., 2020; Manafzadeh and Gatesy, 2021; Richards et al., 2021; Wiseman et al., 2022b; Yamaguchi and Zajac, 1989). Yet, an unconstrained inclusion of joint translations to ROM simulations may lead to an extreme expansion of the estimated mobility beyond its true boundaries. Therefore, such simulations may no longer be functionally informative without the incorporation of additional constraints on joint motion, such as cartilage, muscles and/or ligaments (Arnold et al., 2014; Griffin et al., 2022; Hutson and Hutson, 2012; Jones et al., 2021; Manafzadeh and Padian, 2018; Regnault et al., 2021).

Previous attempts to constrain joint mobility estimates have incorporated ligamentous constraints using the dynamic *nCloth* for simulations in Autodesk Maya (Autodesk Inc., San Rafael, CA, USA; Griffin et al., 2022; Manafzadeh and Padian, 2018). Ligaments were simulated as a dynamic polygon surface, represented by the *nCloth*, wrapping around the bone surfaces from origin to insertion. The length of a ligament was then measured along the midline of this dynamic polygon surface (Baier, 2012; Manafzadeh and Padian, 2018). However, these measurements, and thus the resulting soft tissue constraints, suffered from motion-dependency issues, because the dynamic *nCloth* object is inherently motion dependent. The calculated length of such a ligament is, therefore, highly dependent on the previous timestep, which may cause vastly different length estimates for an identical joint pose depending on different motion trajectories that lead to that pose, necessitating extensive sensitivity analyses (Griffin et al., 2022). This uncertainty may be further amplified if the motion (for which the ligaments are simulated) is non-sequential and includes large jumps in joint angles (e.g. if it is not directly derived from smooth experimental motion data, but based on simulations of joint mobility; see Demuth et al., 2020; Griffin et al., 2022; Manafzadeh and Padian, 2018).

Here, we present a solution to these challenges via implementation of computationally fast approaches for both dynamic, contact-based ROM simulations and motion-independent ligamentous constraints for the study of vertebrate joint function. Our method is integrated within an existing pipeline for joint mobility analyses with the goal of improving joint mobility estimates from functional simulations of extant and extinct vertebrates.

As a case study, we apply our approach to investigate ROM in the avian shoulder. Specifically, we examine unique glenoid of extant birds (Jenkins, 1993) using *in silico* simulations in conjunction with *ex vivo* data collected through X-ray reconstruction of moving morphology (XROMM; Brainerd et al., 2010; Gatesy et al., 2010). Previous work on birds and their ancestors has mainly focused on the hindlimb and terrestrial locomotion (Kambic et al., 2014, 2017; Manafzadeh and Gatesy, 2021; Manafzadeh and Padian, 2018; Manafzadeh et al., 2025; Turner et al., 2020), with others only investigating specific behaviours in the forelimbs (e.g. Baier and Gatesy, 2013; Baier et al., 2013; Heers et al., 2018; Voegele et al., 2022). However, clarifying overall joint mobility within the avian shoulder will be pivotal for improving inferences of wing function during the theropod-to-bird transition, which witnessed the evolutionary origin of avian powered flying ability – one of the most dramatic locomotor transitions in the evolutionary history of vertebrates.

## MATERIALS AND METHODS

### Study species

Five carcasses of red-legged partridges (*Alectoris rufa* Linnaeus 1758; henceforth abbreviated as RLP) were obtained from a local gamekeeper (Table 1). Four specimens were selected for marker placement and experimental data collection (RLP1–4) and an additional specimen (RLP5) was partially dissected to expose ligaments within the shoulder joint for measurement. The specimens were acquired frozen and were thawed for marker implantation (RLP1–4) or dissection (RLP5). The specimens were refrozen after marker placement for CT scanning and storage until they were thawed again for experimental data collection. This work did not require ethical approval from an animal welfare committee.

**Table 1.** Red-legged partridge data collection

Specimen	Body mass (g)	Sex	Marker placement	Trials captured	Trials useable	Dissection data	Freeze–thaw cycles
RLP1	484	M	X	–	–	–	1
RLP2	527	M	X	7	2	–	2
RLP3	492	M	X	7	7	–	2
RLP4	420	F	X	8	2	–	2
RLP5	421	F	–	–	–	X	1

Age at time of death of the individual specimens was not known, but the birds were considered adults based on body masses and skeletal maturity (see Pepin, 1985). Damage to the pectoral girdle and wings of RLP1 was identified in the μCT scan and the specimen was discarded prior to conducting experiments.

### Marker placement and dissection

To ensure precise 3D measurements of joint movement, we implanted radio-opaque markers directly into bones (Brainerd et al., 2010; Brocklehurst et al., 2025; Kambic et al., 2014, 2017; Manafzadeh, 2020; Regnault et al., 2021; Wiseman et al., 2022a). Before marker implantation the specimens were visually inspected to ensure that no effects of desiccation or decomposition were evident, and to assess potential damage to the wings. Small incisions into the skin of four partridge specimens (RLP1–4) were made to expose the surface of the major bones of the pectoral girdle and wing. Subsequently, 1.1 mm holes were drilled into the exposed bones using a small hand-held drill, into which the radio-opaque 1.0 mm tantalum beads (Bal-Tec, Los Angeles, USA) were placed and fixed with cyanoacrylate glue, which was also used to close the incisions. In total, 20 beads were implanted into the pectoral girdle and the bones of one wing for each specimen. Each long bone in the wing (humerus, ulna, radius and carpometacarpus), as well as the scapula and sternum, had rigid bodies defined by three tantalum beads (Fig. S3). Unfortunately, owing to the large amount of musculature and other soft tissue surrounding the coracoids it was not possible to implant the minimum number of markers required to accurately track their position (Manafzadeh, 2020). Therefore, the position and orientation of the coracoids were approximated for the tracking (see Demuth et al., 2023; Wiseman et al., 2022a). Owing to potential coracosternal movement (see Baier et al., 2013) only a dynamic plane could be defined, based on the left and right coracoid markers and the cranial sternum marker (Fig. S3), rather than a rigid body.

**Fig. 1.**
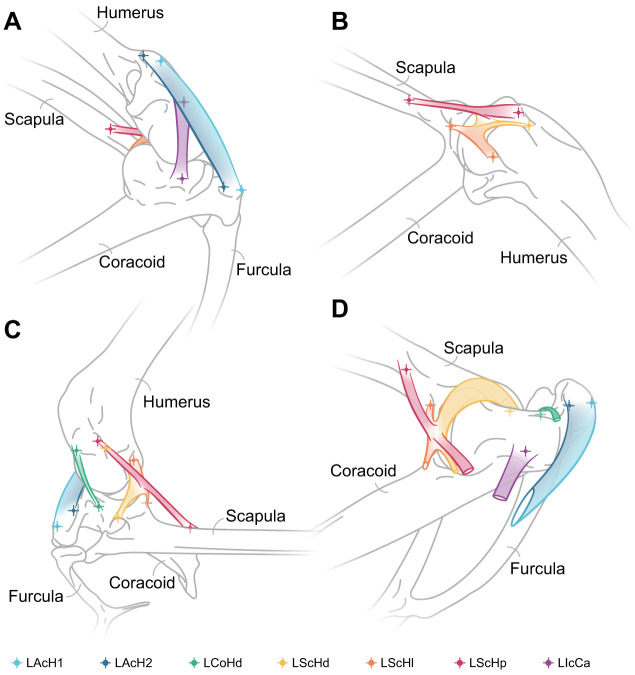
**Shoulder ligaments in the red-legged partridge (*Alectoris rufa*).** Ligaments identified during the dissection of the shoulder capsule of the red-legged partridge. Right shoulder joint in the following views: craniolateral (A), caudolateral (B), dorsal (C) and oblique lateral with removed humerus and cut ligaments (D). Musculature and other soft tissues have been omitted from the illustrations to highlight the ligaments and the positions of their attachment sites on the scapula, coracoid and humerus. Owing to the broad attachment surfaces on the humerus and coracoid, the LAcH was divided into two separate units, 1 and 2. Crosses denote the positions of locators for ligament origins and insertions in Autodesk Maya for subsequent ligament simulations. Note the fusion of the LScH ligaments. Nomenclature follows Fürbringer (1888). LAcH, *ligamentum* (*L.*) *acrocoraco-humerale*; LCoHd, *L. coraco-humerale dorsale*; LScHd, *L. scapulo-humerale dorsale*; LScHl, *L. scapulo-humerale laterale*; LScHp, *L. scapulo-humerale posterius*; LIcCa, *L. intracapsulare coracoideus anterius*.

During dissection of RLP5, the origins and insertions of six shoulder ligaments were identified (Fig. 1) and verified with reference to published literature (Fürbringer, 1888; Razmadze et al., 2018; Watanabe et al., 2021). The lengths of each of these ligaments were measured using digital callipers sensitive to 0.1 mm.

### *Ex vivo* XROMM data collection

*Ex vivo* movement data of the shoulders of RLP2–4 were captured via biplanar fluoroscopy using XROMM (Brainerd et al., 2010; Gatesy et al., 2010; Manafzadeh, 2020). Damage to the wings and pectoral girdle of RLP1 was identified in the μCT scan and the specimen was discarded prior to conducting experiments. The remaining partridges (RLP2–4) were placed on top of a foam board and strapped into a custom-built Velcro harness to secure their position in the capture area of the fluoroscopes. The carpometacarpus/wing was attached to a wooden manipulator rod using Velcro strips (Fig. S1). The Velcro harnesses on both the body and wings and the rod itself allowed for some flexibility to reduce the risk of injuries to the joint capsules due to potentially excessive movement of the manipulator rod while attempting to achieve maximal excursion of the wing. Maximal possible movement was assumed when further excursion was met with resistance (e.g. rolling of the body). Further movement in that direction could have compromised the integrity of the joint capsule and the surrounding soft tissues (see below).

Two BV Libra C-arm systems (Koninklijke Philips N.V., Amsterdam, The Netherlands) were used, each composed of a BV 300 generator, F017 tube and BV 300 collimator and intensifier (22.9 cm diameter), with a source-to-image distance of 99.5 cm. The X-rays were generated at 67 kV and 2.27 mA and fired continuously for the duration of a trial. The trials were recorded with two Photron FASTCAM Mini WX50 high-speed digital video cameras (Photron, Tokyo, Japan) at 250 frames per second with a resolution of 2048×2048 pixels and a shutter speed of 750 Hz. In total 22 trials were recorded approximating 8 min of motion data (Table 1). X-ray videos were undistorted, calibrated and digitised using XMALAB 2.1.0 (Knörlein et al., 2016). The 3D point data of the markers were filtered at 10 Hz prior to export.

### Skeletal geometry acquisition and anatomical and joint coordinate system setup

The specimens were μCT scanned at 190 kV and 200 μA, with a voxel size of 0.125 mm, at the Cambridge Biotomography Centre, Cambridge, UK, using a Nikon 49 Metrology XT H 225 ST scanner to capture both the relative marker positions and the bone geometries. μCT data were segmented using Avizo Lite 2020.2 (Thermo Fisher Scientific, Inc., Waltham, Massachusetts, USA) and the resulting meshes prepared for data analysis and simulations using MeshLab 2021 (Cignoni et al., 2008; Clark et al., 2023).

Anatomical coordinate systems (ACSs) and joint coordinate systems (JCSs) were created following established primitive shape-fitting protocols (Bishop et al., 2021; Demuth et al., 2020; Gatesy et al., 2022). The articular surfaces of the glenoid and wing bones were first isolated in Autodesk Maya and primitive shapes were fitted in MATLAB R2023b (Bishop et al., 2021; The MathWorks, Inc., Natick, Massachusetts, USA; see Supplementary Materials and Methods for detailed description of the ACS setup). Based on the ACSs, the wing was collapsed into a reference pose (Fig. S2) from which all translational and rotational deviations were measured (Baier et al., 2013; Bishop et al., 2021; Gatesy et al., 2010, 2022; Heers et al., 2016; Kambic et al., 2014).

### *In silico* ROM simulations and ROM mapping

Range of motion (i.e. joint mobility) simulations were set up in Autodesk Maya 2025 using a novel Python implementation of the approach originally outlined by Marai et al. (2006) and subsequently implemented in MATLAB by Lee et al. (2023), adapted for Autodesk Maya to incorporate it into existing ROM pipelines (Manafzadeh and Gatesy, 2020, 2022; Manafzadeh and Padian, 2018; Manafzadeh et al., 2021). Our implementation builds on the theoretical framework of previous joint sampling techniques (Manafzadeh and Gatesy, 2020, 2022; Manafzadeh and Padian, 2018; Manafzadeh et al., 2021), yet has significant advantages over these approaches. Importantly, it no longer assumes a fixed joint centre position, and joint translations are optimised for each rotational joint pose. As such, it represents physiological joint movement more accurately by enabling continuous contact of articular surfaces and constant joint spacing, thus overcoming previous methodological constraints. Additionally, our simulations exploit the signed distance field (SDF) representation of the bones (i.e. as a 3D scalar field specifying the signed distance from any given field point to the bone surface; see Lee et al., 2023; Marai et al., 2004, 2006). This only needs to be calculated once, rather than continuously relying on computationally expensive 3D meshes; as such, our method overcomes previous computational constraints, substantially improving simulation speed.

SDFs were initialised as a cubic grid centred around the centroids of the fitted shapes to their respective articular surfaces. The signed distances were then sampled at regular intervals over the 3D grids (*n*=101×101×101 grid points each). For each grid point *P*, the distance *d_P_* to the bone mesh was calculated by finding the closest point *Q* on the surface of the mesh. The sign, distinguishing whether the point *P* was inside (negative) or outside (positive) a bone mesh, was determined by calculating the dot product between the vector 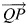 and the surface normal 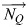 at point *Q*. If the dot product was negative, then *P* was inside the mesh and thus its distance negative:
(1)

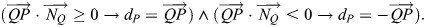


The exact signed distance to the bone surface was, therefore, known only at each grid point. The distance to the bone surface between grid points was interpolated on-demand from the surrounding grid points using tricubic interpolation (Lekien and Marsden, 2005):
(2)

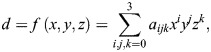
where *a*_*ijk*_ are the coefficients for each of the eight corner points of a grid node (Lekien and Marsden, 2005) and *x*, *y* and *z* are the Cartesian 3D coordinates of the sampled point.

We implemented a multiterm objective function *C*_ROM_ to optimise the translational joint position for each given joint orientation:
(3)

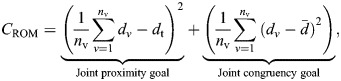
subject to
(4)

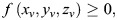
where *d_v_* is the calculated signed distance for vertex *v*, *n*_v_ is the number of vertices of the articular surfaces, *d*_t_ is the target joint proximity and 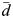 is the mean joint proximity, following Lee et al. (2023). Additionally minimising the variance of the joint proximity ensures maximal congruence between the articular surfaces of the proximal and distal bones, especially in non-spherical joints, such as the particularly open hemi-sellar avian shoulder joint (Griffin et al., 2022; Jenkins, 1993; Sy, 1936). This optimisation problem was solved using a sequential quadratic programming method (SQP; Kraft, 1988) and implemented in Python 3.11 for Autodesk Maya 2025 using the Python modules NumPy 1.24.4 (Harris et al., 2020), SciPy 1.15.0 (Virtanen et al., 2020) and pytricubic 1.0.4 (https://github.com/danielguterding/pytricubic); see Supplementary Materials and Methods.

Joint orientations were sampled across the flexion/extension (FE; −180 deg to 180 deg), abduction/adduction (ABAD; −90 deg to 90 deg) and long-axis (LAR; −180 deg to 180 deg) axes at 5 deg intervals, resulting in 197,173 tested joint orientations per specimen. As the joint centre position was optimised for each rotational pose, the resulting simulation inherently described six DOFs with an optimal joint translation for any given rotational pose, while simultaneously avoiding potentially excessive disarticulations of the joint (Manafzadeh and Gatesy, 2021, 2022).

### Motion-independent soft tissue simulations

Furthermore, we implemented a motion-independent solution for soft tissue simulations into the existing pipeline of ROM simulations in Autodesk Maya, to circumvent motion-dependency issues related to the dynamic *nCloth* object. To do so, the path minimising algorithm of Marai et al. (2004) was implemented in Python for Autodesk Maya 2025 to calculate the length of a ligament at any given joint pose independent of the previous time step. The 3D geometries of each bone were again approximated as SDFs. The ligament path and length could then be optimised over the much faster interpolated scalar field representation of the bones rather than relying on their computationally expensive 3D meshes (Marai et al., 2004).

Individual ligament coordinate systems were defined to facilitate the calculation of the wrapping around the bones. A vector from the origin *p*_0_ to insertion *p_n_* represented the *X*-axis, which was subsequently subdivided into (*n*=20) equidistant segments between *p*_0_ and *p_n_*. The *Y*-axis was defined by the cross product of the *X*-axis and a vector from the origin *p*_0_ to the joint centre *p_j_*, with the *Z*-axis perpendicular to both *X*- and *Y*-axes. The ligament path could then be formulated as the following optimisation problem: find the coordinates of the *n*–1 points between *p*_0_ and *p_n_*, so that the Euclidean distance of the path along *p*_0_, *p*_1_, *p*_2_,…, *p_n_* is minimal while the distance between each path point and the bony obstacles is non-negative. Because the points were equally spaced along the *X*-axis (i.e. their distance was constant) the length of the shortest path could be calculated by minimising its Euclidean distance over only the *y* and *z* coordinates for each point (Marai et al., 2004), which we implemented as the following cost function:
(5)

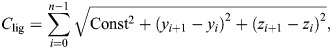
subject to
(6)

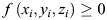
and
(7)

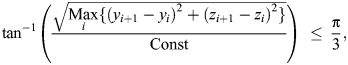
where
(8)

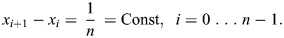
The additional constraint function (Eqn 7) was implemented to ensure the smoothness of the optimised ligament paths and to prevent the ligaments from abruptly changing direction or intersecting the bone meshes between the path points. Hence the angular offset of any two subsequent ligament points could not exceed 60 deg relative to the ligament long-axis.

As with the ROM simulations, the above optimisation problem was solved using SQP (Kraft, 1988); see Supplementary Materials and Methods.

### Mobility simulation comparison

Resultant ligament lengths for each joint pose of the XROMM trials and ROM simulations were exported and subsequently analysed in MATLAB 2023b. Outliers in estimated ligament lengths (i.e. instances in which the SQP optimiser failed to converge) were automatically detected and interpolated from the surrounding sine-corrected rotational data (Manafzadeh and Gatesy, 2020) using *scatteredInterpolant*. The resulting ligament lengths were compared with scaled dissection data and with the maximally sustainable strain observed in previous experimental data (Baier, 2012). The ROM ligament data were additionally compared with maximal length estimates obtained from the XROMM data. Joint poses in the ROM simulations for which any of the six simulated ligaments exceeded the strain tolerances were deemed impossible and, therefore, discarded from the osteological ROM dataset, as the ligaments would either have prevented the bones from attaining such a pose or would have torn. The resulting *in silico* soft tissue-constrained mobility estimates were then compared to the *ex vivo* mobility as determined through XROMM. Volume measures, representing the total movement envelope of a joint (Brocklehurst et al., 2022; Lee et al., 2023; Manafzadeh and Gatesy, 2020; Richards et al., 2021), were determined through fitting of an alpha shape. Because the volume is highly dependent on the alpha value (Brocklehurst et al., 2025; Lee et al., 2023), we conducted sensitivity analyses to determine the optimal values for *ex vivo* and *in silico* data (Fig. S7).

To constrain the ROM simulations, we estimated maximally sustainable strain rates for the soft tissues in multiple ways. Since the estimated ligament lengths are dependent not only on rotational but also translational degrees of freedom, we created two main datasets. Firstly, the maximal ligament lengths present in the XROMM data were determined and directly used as the threshold for the XROMM set. For the ROM set, the ligaments lengths were interpolated using the XROMM data rotational information while retaining the underlaying translational information from the optimised joint articulation, hence their lengths differ from the XROMM data. Additionally, the dissection data for RLP5 were scaled to each individual specimen and an additional strain factor of 26% was applied based on the mean failure strain in experimental data of the acrocoracohumeral ligament in pigeons (Baier, 2012).

## RESULTS

### *Ex vivo* joint mobility

Overall, 11 of 22 recorded trials were useable and fully analysed (RLP2, 2 trials; RLP3, 7 trials; RLP4, 2 trials; Table 1; Fig. S4), approximating a total of 4 min of high-resolution motion data, resulting in 60,027 individual positions and orientations per bone. Eleven trials (50% of the collected data) had to be discarded due to suspected damage of the shoulder joint capsule; for both RLP2 and RLP4 potential damage occurred after two trials (Fig. S4), evident from excessive joint translations. The subsequent trials for these specimens were thus discarded from the shoulder dataset and any subsequent analyses (see Manafzadeh, 2020).

#### Rotational movement

**Fig. 2.**
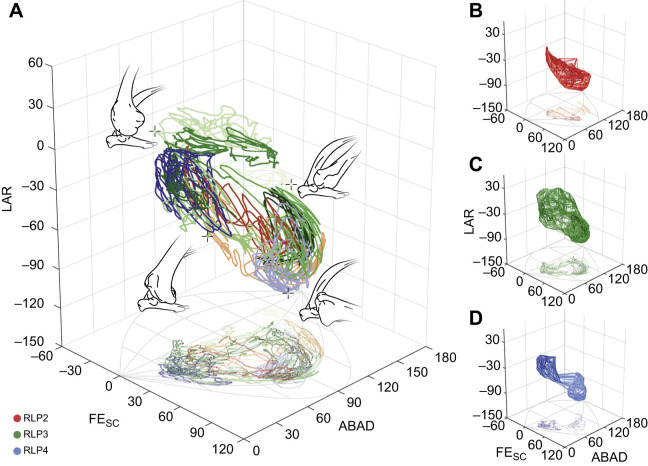
***Ex vivo* rotational mobility of the different red-legged partridge specimens.** (A) Combined and individual rotational joint mobility (B, RLP2; C, RLP3; D, RLP4) of the different specimens in the sine-corrected (SC) space of the two-neutral spherical rotation system (Lee et al., 2023). Illustrations show a selection of representative joint orientations of the humerus relative to the scapulocoracoid with their corresponding data points denoted by crosses. Individual trials are colour-coded from dark to light for each specimen. FE, flexion/extension; ABAD, abduction/adduction; LAR, long-axis rotation.

The three specimens showed similar constraints on joint mobility from their cadaveric manipulation, with their mobility envelopes mostly overlapping (Fig. 2). Differences in captured overall excursion were relatively minor, with the specimens agreeing on individual mobility boundaries. Only two trials were collected prior to joint capsule damage for RLP2 and RLP4; thus, their overall ROM observed (147,265.4 deg^3^ and 89,676.7 deg^3^ for RLP2 and RLP4, respectively) was substantially smaller than that of RLP3 (375,437.8 deg^3^). Movement with positive LAR values (as evident in RLP3) was not captured for neither RLP2 nor RLP4 before damage to the joint capsule occurred, hence this area is ‘unexplored’ in the experimental data of these two specimens; however, it should certainly have been achievable. These missing areas are, therefore, caused by a sampling artefact due to the low number of usable trials, rather than representing true intraspecific variation in joint movement. Owing to the bulk of their individual ROM extent lying within the range seen in RLP3, these two specimens extended the combined XROMM volume to 539,327.3 deg^3^. The sine-corrected flexion/extension ROM of the combined taxa was almost exclusively constrained to positive values (i.e. movement with a laterally oriented humerus, as negative values indicate a medially directed humerus; Fig. 2).

#### Translational movement

**Fig. 3.**
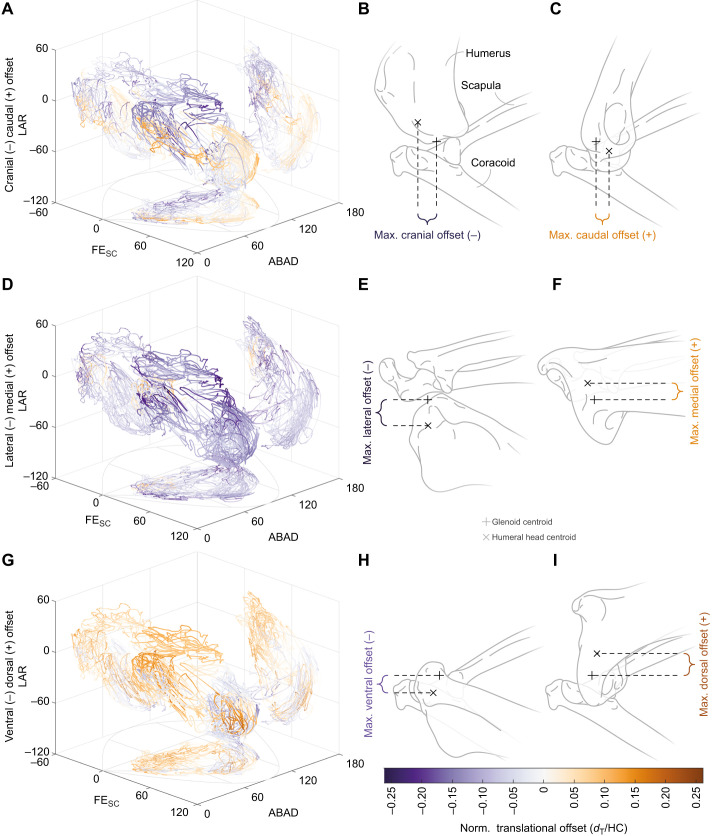
***Ex vivo* translational joint mobility in the red-legged partridge shoulder joint.** Size-normalised craniocaudal (A–C), mediolateral (D–F) and dorsoventral (G–I) offsets between the humeral head and glenoid centroids. Individual rotational poses are colour-coded by the corresponding values of respective translational DOFs. Illustrations (B,C,E,F,H,I) demonstrate shoulder poses with the maximal translational offsets in lateral (B,C,H,I) and dorsal (E,F) views. Red-legged partridge shoulder mobility indicates only limited coupling between rotations and translations across their range of motion, as large translational deviations were not restricted to specific rotational areas and vice versa. *d*_T_, translational displacement; HC, humeral circumference.

Cadaveric manipulation revealed substantial sliding (i.e. translational movement) in the red-legged partridge shoulder joint (Fig. 3; Fig. S5). Owing to the large magnitudes of joint translations, the superimpositions of the ACSs (as determined by the centroids of the fitted shapes to the glenoid and humeral head articular surfaces) were a poor approximation of the functional joint centre (Fig. S6), unlike what has been suggested for other joints, such as the hip joint of other archosaurs (Demuth et al., 2020) or the hip joint in hominins (Wiseman et al., 2022b).

Substantial interactions within and between translational and rotational DOFs were observed, with certain joint positions exhibiting higher variability in joint orientations than others. Large translational excursions were not limited to specific orientations of the humerus relative to the glenoid (Fig. 3). Rotational and/or translational minima or maxima of individual DOFs were never attained simultaneously, similarly to previous studies (Haering et al., 2014; Kambic et al., 2017; Manafzadeh and Gatesy, 2021). There was no apparent coupling between translational and rotational DOFs in the shoulder joint of the red-legged partridge specimens (i.e. extremes in rotations are not constrained to extremes in translations and vice versa; Fig. 3) and translational extremes were distributed evenly across the rotations, unlike reported for other tetrapods (Bishop et al., 2023; Brocklehurst et al., 2025).

#### Ligament length estimates

**Fig. 4.**
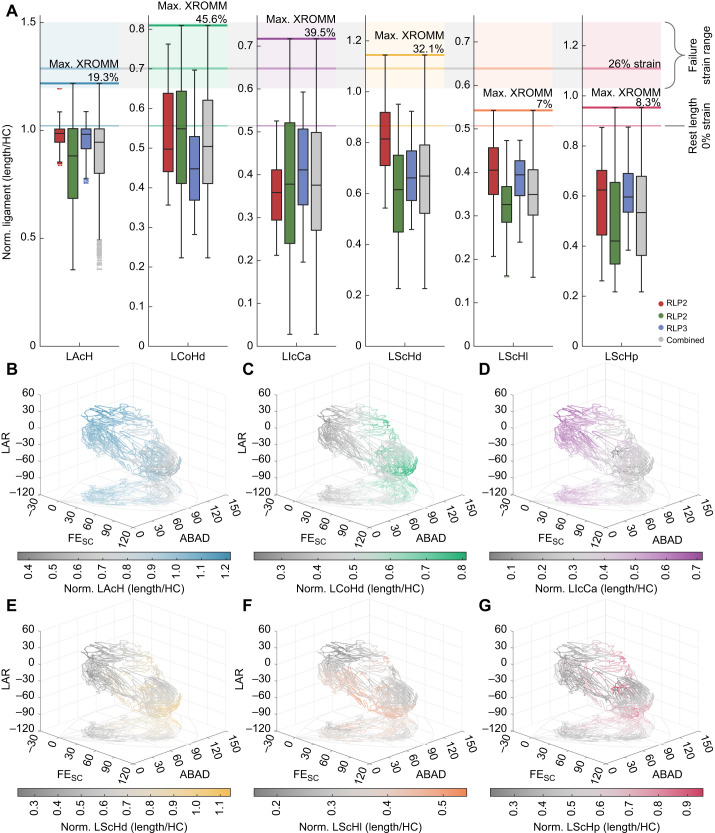
**Normalised ligament length estimates based on XROMM trials.** (A) Comparison between size-normalised ligament lengths from XROMM data and scaled dissection data with experimental failure strain ranges of the acrocoracohumeral ligament (Baier, 2012). (B–G) Normalised ligament lengths calculated from the XROMM data in relation to joint excursion in the sine-corrected two-neutral spherical rotation system. Colours correspond to ligaments in A.

The calculated ligament lengths from the XROMM data approximated the scaled dissection data (Fig. 4; Table S1) for all specimens, while highlighting differences in observed strain ranges among individual ligaments. Ligament length estimates stayed within or below the range of maximally feasible strain in experimental data (17–47%) of the acrocoracohumeral ligament (LAcH; Baier, 2012). The coracohumeral ligament (LCoHd) exhibited the most strain in the cadaveric XROMM data, exceeding the mean failure strain of the LAcH (26%; see Baier, 2012) in both RLP2 and RLP3, with RLP4 remaining just below the threshold. The lateral (LScHl) and posterior (LScHp) scapulohumeral ligaments never reached a taut state during the *ex vivo* experiments in two specimens (RLP3 and RLP4 for the former, and RLP2 and RLP4 for the latter; Fig. 4). The fact that RLP2 and RLP4 never reached a taut state for the LScHp might be due to those specimens' generally smaller ROM extent (Fig. 2) which did not fully cover the total ROM in the shoulder before damage to the joint capsule occurred and, therefore, did not stretch the ligaments to their full extent. The greatest strain range was observed in the intracapsular ligament (LIcCa), with the distance between origin and insertion ranging from 5.4% of resting length (–94.6% strain) to 139.5% (39.5% strain).

The greatest ligament strains were observed in abducted (elevated, ABAD <90 deg) joint poses with positive axial rotation (LAR) for the LAcH and LIcCa, whereas the opposite was true for the LCoHd and the three LScH (adducted/depressed ABAD >90 deg; Fig. 4B–G). Note that the two-neutral spherical transformation does not directly correlate with motion around the cardinal axes because of embedded axial rotations and is thus less intuitive; however, it is able to prevent issues related to gimbal lock and thus preserves relative distances between individual joint poses in 3D (Lee et al., 2023).

### *In silico* joint mobility

#### Osteological ROM

The osteological ROM overestimated the *ex vivo* joint mobility captured via XROMM by an order of magnitude across all specimens (Table 2). The pose space generated by the ROM simulations initially resulted in many osteologically feasible, yet physiologically unlikely, joint poses if soft tissue constraints were considered (e.g. ligaments). However, the optimised *in silico* joint translations approach the translational *ex vivo* kinematics (Fig. S8), indicating that our simulations successfully resemble true articular behaviour.

**Table 2.** Simulation results and comparison with XROMM data

Specimen	Dataset	Volume (deg^3^)	Missed (deg^3^)	Missed (%)
RLP2	ROM	3,550,553.25	30,144.40	5.59
	Dissection	1,573,886.51	162,207.39	30.08
	XROMM	1,919,651.02	92,909.87	17.23
	Full	6,100,591.27	26,407.15	4.90
RLP3	ROM	3,202,769.72	10,413.26	1.93
	Dissection	2,841,991.28	96,699.85	17.93
	XROMM	3,081,498.41	9,337.84	1.73
	Full	5,993,494.98	2,944.38	0.54
RLP4	ROM	3,051,695.43	585.96	0.11
	Dissection	2,048,592.97	129,366.39	23.99
	XROMM	2,212,443.72	79,421.31	14.73
	Full	6,156,200.81	301.59	0.06
All	*ex vivo*	539,327.29	–	–

Ligament strain thresholds used to constrain the ROM volume are in Table S1. Owing to differences in joint translations, different datasets resulted in different ligament length estimates for identical rotational poses. The ‘ROM’ dataset represents the constrained dataset where the ligament lengths have been interpolated across all cadaveric joint orientations but within the ROM simulation dataset (and their underlaying translations); ‘XROMM’ represents the constrained dataset for which the maximal ligament lengths were directly calculated from the *ex vivo* movement (i.e. the XROMM dataset); ‘Dissection’ represents the constrained dataset where the dissection values were scaled by humeral circumference to the corresponding specimen with an additional strain factor of 26% (mean failure strain from experimental data of the acrocoracohumeral ligament; see Baier, 2012); the ‘Full’ dataset represents the unconstrained, osteological ROM dataset. Note that the maximal ligament lengths calculated from the ROM datasets tend to be greater than those from the XROMM datasets (see Table S1), thus resulting in greater volumes.

**Fig. 5.**
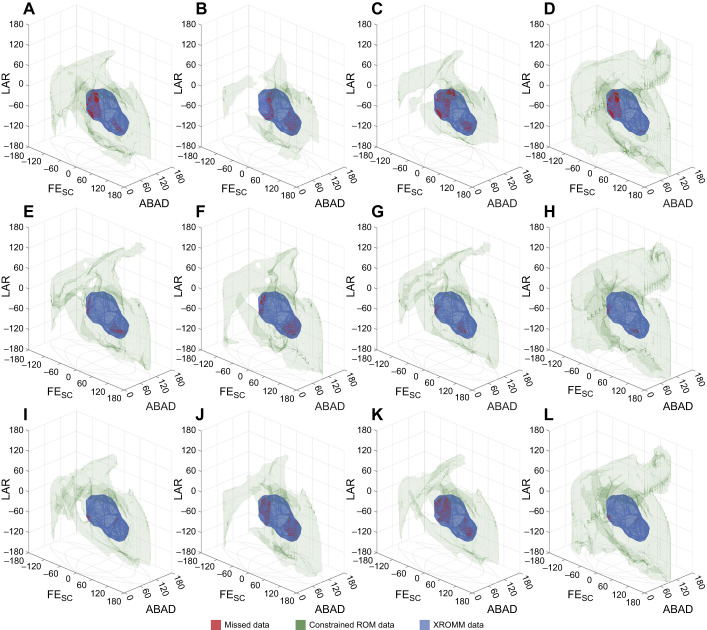
**Comparison between cadaveric joint mobility, osteological and ligament-constrained ROM simulation data.** Specimens are in rows: RLP2 (A–D), RLP3 (E–H) and RLP4 (I–L) and datasets in columns. (A,E,I) ROM-constrained simulation, in which the ligament thresholds were based on the interpolated maximal lengths for each ligament based on the ROM simulation dataset across all XROMM poses. (B,F,J) Dissection data-constrained ROM simulations using scaled and ‘strained’ dissection data of RLP5 (i.e. +26% strain; see Table 2, Table 1). (C,G,K) XROMM data-constrained ROM simulations. (D,H,L) Full osteological ROM without any soft tissue constraints. Red volumes represent missed poses, green volumes are the constrained ROM simulations and blue volumes are the combined *ex vivo* joint mobility. See Table 2 for measurements and details on the individual datasets.

The osteological ROM simulations of all specimens failed to capture portions of the experimental movement envelopes, especially joint poses with a combination of negative FE_SC_ and LAR angles (Fig. 5A), missing from 585.96 deg^3^ (RLP4) to 26,407.15 deg^3^ (RLP2), respectively representing 0.06% and 4.90% of the total range of cadaveric joint mobility. While the full range was not recovered in these osteological simulations, the missing areas were predominantly located at the border of cadaveric joint mobility and may represent an artefact of the sampling resolution, especially in RLP3 and RLP4, where the missing areas remain below 1%. The ROM simulations were sampled at 5 deg intervals (Manafzadeh and Gatesy, 2022; Manafzadeh and Padian, 2018) owing to a trade-off between accuracy and processing time, as joint poses and the resulting processing time both scale exponentially (Bishop et al., 2023; Manafzadeh and Gatesy, 2022) with a decreasing sample interval (on average 0.613 s per joint pose for a random subset of 10,000 joint orientations on an Apple M2 Pro @3480 MHz on a single core). Given the small percentages of missed data in those two specimens (<1%), any missed joint poses would almost certainly have been recovered with a higher sampling resolution and are thus deemed negligible. Decreasing the sample interval (and thus exponentially increasing the number of joint poses) would have come at the expense of impractical computational runtimes for these simulations.

#### Ligamentous constraints on mobility

The constraints imposed by the simulated ligaments substantially reduced osteological joint mobility estimates (Fig. 5). While the scaled and ‘strained’ dissection data yielded the least mobility overall, it was a poor approximation of the *ex vivo* mobility owing to missing substantial portions of cadaveric movement (missing 17.93–30.08% of total *ex vivo* joint mobility; Table 2).

Differences in the missed volumes between the full osteological ROM and the ROM-constrained estimates (i.e. the maximal interpolated values from the ROM ligament dataset across all XROMM coordinates) were relatively minor (<1.5%), only marginally increasing the number of missed poses (Table 2), whereas the soft tissue constraints substantially reduced overall joint mobility by 47.1% (RLP2), 39.3% (RLP3) and 32.6% (RLP4). Owing to differences in joint translations between the XROMM poses and the optimised ROM positions, the calculated maximal ligament lengths for the latter often exceeded the former (except for LScHp in RLP2; see Table S1). Therefore, the XROMM-constrained ROM estimates often missed substantially more cadaveric joint mobility than the ROM-constrained estimates, apart from the case of RLP3, for which the XROMM-constrained mobility estimates missed less of the *ex vivo* poses than the ROM-constrained estimates.

## DISCUSSION

### Importance of the absence of a functional joint centre

The centroids of either shape fitted to the articular surfaces failed to predict the shoulder joint centre position accurately, even though the mean joint centre was mostly positioned within these shapes (Fig. S5). This has fundamental implications for reconstructing joint mobility of extinct taxa. Considering that extensive translational movement is evident in the bird shoulder (Baier, 2012; Jenkins, 1993; Sy, 1936) and present in other animal joints (Brocklehurst et al., 2025; Tsai et al., 2019), the functional (mean) joint centre position cannot be reliably inferred from the preserved bony articular surfaces via shape-fitting. Primitive shape fitting is a useful tool for standardising joint and bone orientations (Bishop et al., 2021; Gatesy et al., 2022); however, the resulting joint axes and joint centre position may not necessarily represent the true functional joint axes or their centroids for complex joints, but rather a standardised reference frame enabling comparisons among multiple specimens. Translational DOFs must, therefore, be included in any such comparison of joint mobility estimates (Manafzadeh and Gatesy, 2021), otherwise the exclusion of possible but missed joint poses may result in erroneous conclusions and/or functional inferences. In a joint as open as the avian shoulder joint (Jenkins, 1993; Sy, 1936), translations need to be considered to obtain accurate mobility estimates (Baier, 2012; Griffin et al., 2022; Manafzadeh and Gatesy, 2021; Wiseman et al., 2022b). Therefore, joint mobility simulations using only a single joint centre (or multiple rigid ones) are inadequate for accurate estimates of joint mobility. Only truly dynamic joint centres, constrained via consistent articular congruence as presented here (or similar approaches presented elsewhere; e.g. see Bishop et al., 2023; Lee et al., 2023; Manafzadeh et al., 2024), enable accurate osteological mobility estimates.

### Soft tissue influences on avian shoulder joint function

The avian shoulder joint is subject to large forces during flight which are mitigated by a suite of ligaments that prevent dislocation of the joint during movement (Baier et al., 2007; Jenkins, 1993; Sy, 1936). Whereas the LAcH is the main ligament preventing ventral dislocation of the shoulder, counteracting and transmitting the pulling force of the pectoralis muscle through the strut-like coracoids (Baier et al., 2007), it also secondarily restricts excessive abduction of the humerus in combination with the LIcCa. Excessive adduction of the humerus is prevented by the LCoHd and the LScHd, and those ligaments additionally support the suspension of the humerus during the down stroke, whereas the LScHl and LScHp prevent excessive extension/protraction. As with most collateral ligaments, the avian shoulder ligaments do not only prevent excessive rotational movements but also translations beyond natural joint mobility and ensure that the joint surfaces stay in articulation during the considerable sliding movements present in the avian shoulder joint. The osteologically unconstrained and open hemi-sellar morphology of the avian glenoid (Jenkins, 1993; Sy, 1936) differs in orientation from other (quadrupedal) tetrapods, yet shows little difference in absolute shoulder joint mobility in comparison with other reptiles that possess hemi-sellar glenoids, such as tegu lizards (*Salvator*; Brocklehurst et al., 2025) or crocodilians (*Crocodylus*; Pierce et al., 2012). However, their joint mobility differs from the more constrained shoulder joints of non-mammalian synapsids (screw-shaped in *Dimetrodon*; Bishop et al., 2023) and mammals (hemi-sellar joint of the monotreme *Echidna*; Regnault and Pierce, 2018; Regnault et al., 2021; and the ball-and-socket joint of therians; Brocklehurst et al., 2025) for which joint stability is more crucial and constraints are necessitated to support the animal's body weight. These observations provide further evidence that hemi-sellar joints are not generally less mobile than ball-and-socket joints, but use translations to accommodate joint excursions. In our experiments, the hemi-sellar glenoid's articular cartilage constrained translational movement mostly to an axis perpendicular to it (Fig. 3; Fig. S5), as was hypothesised by Sy (1936), increasing rotational movement of the humerus during flapping motion (Fig. S9), whereas the ligaments facilitate constant contact within the shoulder capsule.

Individual ligaments appear to show differences in their elasticity (Fig. 4), with the LAcH being relatively stiffer than the other five, with the observed strain remaining below 20% in all specimens, whereas the LScHd, LIcCa and LCoHd all exceeded 30% strain during cadaveric movement. This could indicate potential differences in the composition and mineral content of these ligaments (e.g. see Landis et al., 1995) and that some may be able to passively store and release elastic energy for usage during the wing stroke. Tendons, which store and release elastic energy (acting like springs) play an important role in terrestrial (Alexander and Bennet-Clark, 1977; Cox et al., 2019; Henry et al., 2005) and aerial (Konow et al., 2015) locomotion. Energy is transferred into a tendon by inertial forces (i.e. deacceleration, such as when the limb is flexed), which is then released through tendon recoil to power the extension of the limb. Such energy storage has been proposed in the humerotriceps (Berg Robertson and Biewener, 2012) and supracoracoideus (Tobalske and Biewener, 2008) muscle-tendon units in pigeons, for which a phase-lag has been observed between fascicle length change and wing joint motion, indicating that stretch and recoil of their tendons may assist and/or power wing movement. The contribution of energy by tendons, therefore, plays an important role in flight mechanics. The potential storage and recovery of inertial energy in ligaments within wing joints has not been previously assessed and, thus, deserves future investigation. Furthermore, given that ligaments are an integral component of the force balance within the avian shoulder joint during flight (Baier, 2012), simulating ligamentous forces and calculating their strain could be an important factor in improving musculoskeletal models of avian wing and shoulder function (e.g. Agrawal et al., 2022; Heers et al., 2018; Tang et al., 2025) that has previously been overlooked.

Ligaments are, however, not the only soft tissues influencing joint function. Additional soft tissues, especially muscles, play an important role in active and passive joint stabilisation and preventing excessive and/or harmful movements (Arnold et al., 2014; Hutson and Hutson, 2013; Lee et al., 2020; Pierce et al., 2012). Unfortunately, their contribution and the specific role of individual muscles could not be directly assessed here. However, given the extent to which ligament-constrained ROM estimates exceed cadaveric joint mobility (Fig. 5), their influence must be substantial, affording an avenue for further investigation to better constrain and infer the functional joint mobility within vertebrate joints.

Our results also hold implications for future investigations of the evolution of shoulder ligaments throughout archosaur evolutionary history and the origin of avian flight. Although previous work has investigated the evolutionary and functional implications of individual shoulder ligaments (Baier et al., 2007) and glenoid joint evolution and function (Jenkins, 1993), the evolutionary history of the full complement of major shoulder ligaments throughout archosaur evolutionary history remains unknown, let alone the evolution of the ligaments' functions. This is a fruitful potential direction for future investigations.

### Conclusions

This study represents a significant advance in our ability to implement and infer soft tissue constraints on joint mobility estimates in both living and extinct taxa to overcome some previous challenges set by translational joint sliding. The implementation of the algorithms initially outlined by Marai et al. (2004, 2006) into Autodesk Maya directly integrates them into the most commonly used software package for joint mobility estimation and skeletal animation in non-human vertebrates (Brainerd et al., 2010; Demuth et al., 2020, 2023; Gatesy et al., 2010; Herbst et al., 2022b,c; Jones et al., 2021; Kambic et al., 2017; Manafzadeh and Gatesy, 2020, 2021, 2022; Manafzadeh and Padian, 2018; Nyakatura and Demuth, 2019; Nyakatura et al., 2015; Wiseman et al., 2022a,b; Zwafing et al., 2021), and presents a significant improvement and expansion of the existing pipeline for investigating form–function relationships of vertebrate joints. Joint ROM simulations are no longer limited to physiologically implausible static joint centres but approximate the true behaviour of joints and their articulation using contact-based optimisation. Such simulations intrinsically describe six DOFs in their motion. By simulating soft tissues, osteological ROM can be constrained to a more functionally informative mobility envelope, thus enabling more accurate inferences through the exclusion of physiologically infeasible joint poses.

Understanding the function of the avian shoulder joint is fundamental to understanding the avian wing stroke. Evidently, the bird shoulder cannot be approximated by an idealized ball and socket joint. Indeed, substantial sliding and/or rolling occurs within the hemi-sellar avian shoulder joint in order to achieve certain joint poses (Figs 2,3; Baier, 2012; Jenkins, 1993; Sy, 1936). Thus, hemi-sellar joints are not necessarily less mobile than ball-and-socket joints, but rather employ translations to increase mobility. The reorientation of the glenoid to face dorsolaterally in birds, rather than posterolaterally as in non-avian reptiles, accommodates the avian wing stroke (Jenkins, 1993). The importance of this evolutionary reorientation is reinforced by our findings for the mobility of the partridge shoulder presented here. Reorientation of this joint directly facilitated the large degrees of abduction/elevation evident in our cadaveric joint mobility results (Fig. 2), such that the wing can be raised far above the shoulder. This ability may represent a fundamental adaptation facilitating take off from the ground and the active, flapping flight of many extant birds. Such movement is not permitted with a laterally facing glenoid like that of extant non-avian reptiles (Brocklehurst et al., 2025) and non-avian dinosaurs (Jenkins, 1993).

Our approach will help facilitate justified functional and evolutionary inferences of joint mobility in taxa that are extinct or otherwise impossible to study with *in vivo* data, adding to an increasing methodological repertoire to help researchers decipher articular form–function relationships and their evolutionary history. Simulated soft tissues constrain the overexpansion of osteological mobility estimates caused by sliding within joints through the exclusion of physiologically unfeasible joint poses and approach cadaveric mobility more closely. Our study's methodological innovations will allow functional interpretations of joint mobility data and improve palaeobiological inferences regarding joint form and function throughout the evolutionary history of vertebrates.

## Supplementary Material

10.1242/jexbio.250952_sup1 Supplementary information
